# Drama-in-education in rural Chinese primary schools: effects on self-concept, social connectedness, and psychosocial wellbeing

**DOI:** 10.3389/fpubh.2026.1812030

**Published:** 2026-04-22

**Authors:** Renfei Liu, Yiliu Pu, Peng Cui

**Affiliations:** 1 School of Civil Engineering, Nanjing Forestry University, Nanjing, China; 2 School of Marxism, Nanjing Forestry University, Nanjing, China; 3 Umeå Universitet, Umeå, Sweden

**Keywords:** arts-based intervention, drama-in-education, psychosocial wellbeing, rural primary school children, self-concept

## Abstract

**Introduction:**

Self-concept is a central developmental resource supporting children's psychosocial wellbeing, resilience, and school adjustment. In rural and low-resource educational settings, structured and scalable interventions that promote social connectedness and positive self-identity remain limited. Drama-in-Education (DIE), an arts-based group practice grounded in role-taking and collaborative enactment, has shown promise in enhancing socio-emotional competencies; however, evidence for short, high-intensity implementations under real-world school conditions remains scarce. This study evaluated the short-term effects and mechanisms of a structured DIE program on rural primary school children's self-concept.

**Methods:**

A quasi-experimental mixed-methods design was employed (*N* = 300) in a rural Chinese primary school. Classes were assigned to either a 10-day intensive DIE intervention or a waitlist control group. Children completed the Piers-Harris Children's Self-Concept Scale, Second Edition (PHCSS-2), at baseline (T0) and post-intervention (T1). Intervention effects were analyzed using ANCOVA, controlling for pre-test scores. Qualitative data were collected through semi-structured interviews, classroom observations, volunteer logs, and reflective writings, and analyzed using thematic analysis.

**Results:**

After adjusting for baseline differences, the intervention group demonstrated significantly greater improvements in total self-concept than the control group (*p* < 0.001), with a medium between-group effect (ηp^2^ = 0.051) and a large within-group pre–post effect (*d* = 1.16). Qualitative findings identified three proximal mechanisms: (1) embodied role-taking that fostered competence-based identity reappraisal, (2) emotionally safe spaces that expanded expressive and gender flexibility, and (3) collaborative peer interaction that strengthened belonging and perceived social support.

**Discussion:**

Short, intensive DIE programs may offer a feasible, culturally adaptable, school-based strategy to enhance self-concept and social connectedness in rural contexts. By combining structured role engagement with reflective integration, DIE appears to activate both individual and relational pathways of psychosocial development. Limitations include non-random assignment, single-site implementation, and absence of long-term follow-up. Future research should incorporate randomized designs and longitudinal assessments to evaluate sustainability and scalability.

## 1 Introduction

### 1.1 Developmental foundations of children's self-concept under educational inequality

Children's psychosocial development provides a foundation for lifelong learning, mental health, and social adaptation. Self-concept refers to children's integrated representations of their abilities, worth, and social roles, and it functions as a key psychological resource supporting learning motivation, emotion regulation, and resilience. Yet educational resources remain unevenly distributed globally, and inequities in access and quality persist. As of 2024, an estimated 251 million children and adolescents were out of school, and in low- and middle-income countries approximately 70% of 10-year-olds experience learning poverty (1–3). These realities highlight a shared policy and practice challenge in resource-constrained settings: identifying school-based interventions that are cost-controlled, feasible, and replicable. Evidence from developmental science and the economics of education suggests that high-quality early and in-school interventions can yield long-term returns, particularly when they strengthen children's self-concept and resilience (4).

From a developmental perspective, self-concept and resilience jointly support children's learning and adaptation. Empirical studies indicate that strengthening self-referential capacities such as self-esteem and emotion regulation can promote resilience and subjective wellbeing, and early self-identification is linked to emotional knowledge and socio-emotional development around school entry (5, 6). In Erikson's theory, primary school age corresponds to the stage of industry vs. inferiority (approximately ages 6–12), during which competence-related self-representations are shaped through visible accomplishment and internalized social evaluation; positive experiences at this stage may extend into later emotional health and social adaptation (7). Accordingly, interventions targeting self-concept in primary school are often viewed as a critical pathway for strengthening children's resilience and adaptive capacities.

In China, despite near-universal coverage of compulsory education, rural children continue to face constraints associated with limited resources, household economic pressure, and urban–rural cultural differences. School priorities may remain test-oriented, and systematic mechanisms to cultivate self-concept and social-emotional competencies can be insufficient, potentially undermining the development of competence and belonging (8). For children living in low-resource environments, schools are often the most stable support system. A pressing challenge for child development and educational practice is therefore how to embed feasible psychosocial intervention pathways into routine schooling.

Compared with better-resourced school settings, rural schools may provide fewer structured opportunities for psychosocial support and school-based mental health promotion. Recent evidence from China suggests that school mental health programs remain unevenly distributed across regions, with persistent gaps in resource allocation and insufficient attention to younger children's developmental needs (9, 10). At the same time, the mental health of children is shaped not only by family and individual factors but also by school-level supports, including the availability of psychological courses and supportive teacher–student relationships (11, 12). These constraints make low-cost, school-based interventions especially important for children in rural settings, where self-concept development may depend heavily on opportunities for recognition, participation, and supportive peer interaction (13, 14).

### 1.2 Drama-in-education as a school-based psychosocial intervention for children

Drama-in-education can function as a school-based psychosocial intervention by creating a participatory and emotionally meaningful space for children's expression, interaction, and reflection. Through role-taking, improvisation, and collaborative meaning-making, children may explore self and others in socially situated ways. Research and practice in China have begun to indicate the value of drama-based approaches for children's self-expression and socio-emotional development in school settings (15).Internationally, drama in education has generated program models across literacy, classroom engagement, and mental health, including sustained school-based approaches and classroom integration through practices such as Mantle of the Expert and dramatic inquiry (16, 17). Beyond providing a platform for emotional expression, drama-based learning can cultivate empathy and collaborative behavior (18), and drama-based interventions have been associated with reductions in psychosocial difficulties in specific contexts (19). Emerging intervention studies have also linked drama-based participation to improvements in emotional understanding and broader psychosocial adjustment in educational settings (20, 21).

Mechanistically, drama activities couple emotion, movement, and language within shared tasks, creating a natural context for embodied learning. Recent review evidence suggests that participatory arts-based and school-based drama approaches may support socio-emotional development through play, embodied understanding, imaginative engagement, empathy, and perspective taking. Recent work on school-based dramatherapy further suggests that the creation of safety, both in the therapeutic relationship and in the activity space, together with the development of meaningful social connections, may be among the key active ingredients that help children engage, communicate, and reinterpret personal experience (22).A mixed-studies review indicates that embodied approaches can reliably improve classroom participation and language output, providing a plausible mechanism for process drama and drama-based literacy (23). In early childhood samples, drama-based activities designed to strengthen emotional intelligence have been shown to improve emotion recognition, prosocial behavior, and social skills, suggesting proximal effects on emotion-related and social outcomes (24). As a group-based practice, drama in education may also have broader relevance for inclusive and equity-oriented education, given emerging evidence on social communication, cooperation, and empowerment-oriented pedagogy across diverse educational contexts (25–28).

### 1.3 Theoretical pathways linking drama-in-education to children's self-concept

Building on Erikson's emphasis on industry, competence, and recognition, drama-in-education may be especially relevant during middle childhood, when children's self-evaluations become increasingly tied to what they can do, how they are appraised, and whether they feel effective in school. Recent longitudinal evidence shows that school-based basic psychological need satisfaction predicts higher self-esteem, while developmental work in the early school years links ability self-concept closely to intrinsic value and performance. Experimental research further suggests that feedback can directly shape children's self-concept through achievement-related emotions (29–31).

From a social-interactional perspective, children's self-concept is therefore not formed in isolation, but through participation, feedback, belonging, and the interpretation of others' responses. Recent research indicates that changes in peer belonging and supportive school climate are associated with more positive emotional health, while school-based creative interventions are valued by parents and teachers because they foster emotional expression, self-esteem, and peer connectedness. This makes role interaction and group feedback theoretically important routes through which drama may shape how children see themselves (32, 33).

From an embodied and dramatic pedagogy perspective, drama is distinctive because it integrates emotion, movement, language, imagination, and collaboration into a shared experience. Recent review and meta-analytic evidence suggests that embodied action can enhance learning processes and outcomes, while classroom-based drama studies show that gesture and body movement can mediate gains in children's narrative performance (34–36).

### 1.4 Research gaps and the aims of the present study

Despite accumulating evidence for benefits of drama in education, three gaps remain salient and directly motivate the present study. First, intervention evidence remains limited among rural primary school populations, and reporting often lacks standardized documentation of local adaptation, implementable components, and fidelity monitoring, constraining comparability. Second, culturally situated, child-appropriate adaptations of canonical texts are under-documented in testable and transferable ways, with limited reporting on how local cultural resources and language practices are integrated into key components. Third, mixed-methods studies frequently present quantitative and qualitative findings in parallel without integration, leaving limited evidence on how changes occur and under what classroom conditions effects are strengthened or constrained.

To address these gaps, the present study implements a short, high-intensity drama in education program within real rural classroom conditions. It administers the PHCSS-2 pre- and post-tests to evaluate change in self-concept, and it triangulates qualitative evidence from observations, student reflections, and interviews to characterize proximal pathways of change. The intervention uses a child-appropriate adapted version of As You Like It and integrates a local intangible cultural heritage element (Dabie Mountain folk songs), with implementation processes documented to support transferability. The study addresses three research questions:

**Question 1:** To what extent does participation in a short, high-intensity drama-in-education program improve rural primary school students' overall self-concept and its related dimensions, compared with a control group?

**Question 2:** How do children experience and narrate changes in self-concept during drama activities?

**Question 3:** What classroom interaction patterns and school-context conditions appear to facilitate or constrain the effects of the intervention?

By addressing these questions, the study aims to offer rural schools an operational and replicable arts-based intervention pathway and to extend the evidence base on drama in education as a means of supporting children's self-concept and resilience. The study does not claim to resolve high-intensity design demands such as randomization and long-term follow-up; instead, it contributes setting-sensitive evidence through contextualized implementation and integrated quantitative–qualitative reporting, while advancing feasible pathways for cultural adaptation in rural school settings.

## 2 Methods

### 2.1 Research design

This study used a quasi-experimental pretest–posttest control-group design to evaluate the immediate effects of a short-term drama in education intervention on rural Chinese primary school children's self-concept. A total of 300 students participated (intervention group, *n* = 150; control group, *n* = 150). The intervention group took part in an intensive drama in education program delivered over 10 consecutive days, whereas the control group continued routine school activities during the same period.

A mixed-methods approach was adopted to integrate quantitative and qualitative data in order to capture both measurable changes in self-concept and the proximal processes through which change may occur. Quantitative data were collected using a standardized self-concept measure administered before and after the program. Qualitative data were drawn from classroom observations, semi-structured interviews, and students' reflective texts.

**Figure 1:**
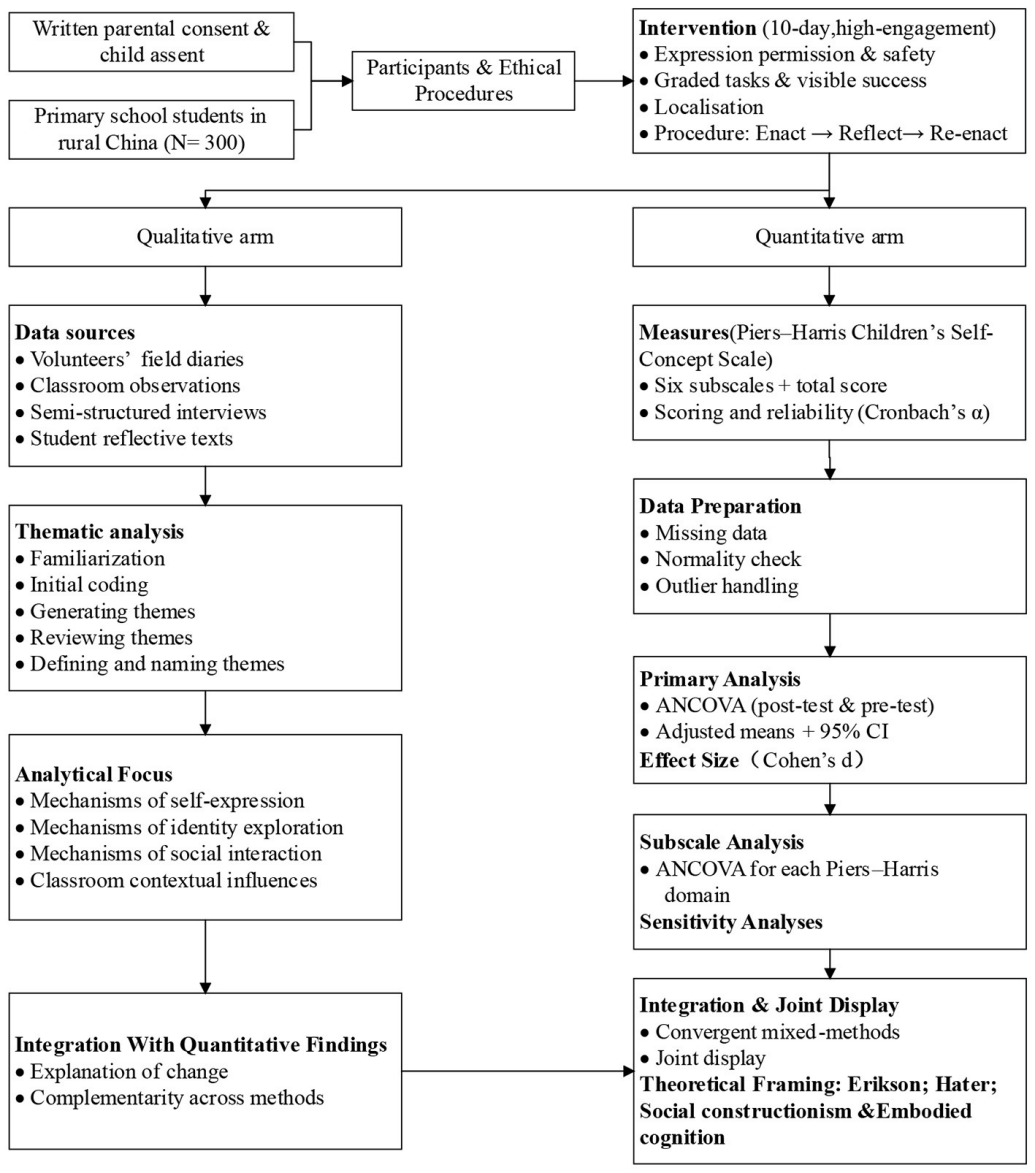
Experimental intervention pathway.

As shown in Figure 1, the study focused on short-term psychological responses—such as self-expression, self-awareness, and social interaction—rather than long-term structural changes in developmental trajectories. This design is informed by developmental and intervention research suggesting that high-density, context-rich experiential programs can generate meaningful psychological effects and short-term fluctuations in cognition and affect within a relatively brief timeframe (37, 38).

### 2.2 Participants and data collection

The intervention was implemented in a resource-limited rural primary school (anonymized) in Anhui Province, China. The school served children from a relatively under-resourced rural community. In total, 300 primary school students participated in the study, with 150 assigned to the intervention group and 150 to the control group.

Participants in the intervention and control groups were recruited from existing classes across the primary school years, specifically from Grades 2 to 5, within the same rural primary school. Because the study adopted a quasi-experimental design, group assignment followed existing class arrangements rather than individual randomization.

Inclusion criteria for the quantitative sample were: (1) enrollment in the participating rural primary school during the primary school years, specifically in Grades 2 to 5; (2) basic language comprehension and expressive ability sufficient to understand classroom instructions and complete the assessment procedures; (3) written informed consent from a parent or guardian; and (4) child assent provided orally or in writing. Exclusion criteria included: (1) anticipated low or unstable attendance during the intervention period, such as long-term absenteeism, planned transfer to another school, or health conditions likely to prevent regular participation; (2) inability to complete the pretest or posttest procedures; and (3) substantial absence from intervention activities or data collection sessions. Written consent procedures were completed for all participating children and their guardians. Permission for the study was obtained from the school administration, and all procedures adhered to ethical standards for research with minors and the principle of data minimization.

To assess intervention effects, the study administered the Piers–Harris Children's Self-Concept Scale, Second Edition (PHCSS-2) (see Appendix S1). The scale includes 80 dichotomous (Yes/No) items and yields six subscale scores—Behavioral Adjustment, Intellectual and School Status, Physical Appearance and Attributes, Freedom from Anxiety, Popularity, and Happiness and Satisfaction—as well as a total self-concept score. A Simplified Chinese version translated and adapted using standard procedures was used.

Both groups completed the PHCSS-2 at two time points: one day before the intervention (T0) and 1–2 days after the intervention ended (T1). Administration was conducted in classrooms by trained research assistants following standardized instructions. Students were informed how to respond, encouraged to answer independently, and assured that responses were for research purposes only and would not be used for individual evaluation. Research assistants responded only to comprehension questions and did not provide interpretive or leading prompts.

Questionnaire data were entered using a double-entry procedure by two independent coders, with discrepancies resolved against the original paper questionnaires. Data were then fully de-identified by replacing names and class identifiers with unique study IDs; the linkage file was stored separately from the analytic dataset. The final dataset contained no missing values, and there were no cases lost to follow-up; all students meeting inclusion criteria and completing both T0 and T1 assessments were included in the quantitative analyses.

To examine proximal mechanisms and contextualize the quantitative findings, multi-source qualitative data were collected throughout the intervention period and immediately after T1 from the intervention group. For the qualitative component, students were purposively selected from the intervention group. Inclusion criteria included completion of the intervention, willingness to participate in interviews or group sharing, and ability to reflect on and communicate their experiences. To capture variation in experiences, selection also considered diversity in gender, classroom engagement, and observed change during the intervention, where applicable. Students who did not complete the program or were unavailable during the qualitative data collection period were not included in the qualitative sample.

Qualitative materials were collected throughout the intervention period and immediately after T1 to capture both proximal classroom processes and children's retrospective accounts of change. These materials included repeated classroom observations (8–10 sessions per class) guided by a semi-structured protocol focusing on self-expression, emotion regulation, collaborative interaction, and role engagement; daily logs completed by classroom volunteers to document participation highlights, critical incidents, and implementation challenges; small-group debrief records facilitated by research assistants after key activities to capture children's immediate reflections; and brief student reflective writings collected at selected time points.

In addition, semi-structured interviews were conducted after T1 with approximately 20 students from the intervention group selected via maximum variation sampling based on gender, grade, classroom participation level, and magnitude of PHCSS-2 change. The interviews focused on children's feelings during drama activities, perceived changes in self-expression, confidence, peer relationships, and memorable moments in the program. Interviews lasted 20–30 min, were audio-recorded, transcribed verbatim, and anonymized using study IDs. Demographic information was reported only in aggregated or stratified form to minimize identifiability. Together, these multiple sources enabled triangulation between observed classroom processes, immediate activity-based responses, and children's retrospective narratives. The interview guide is provided in Appendix S2.

### 2.3 Intervention procedures

The intervention was based on a child-adapted, localized version of Shakespeare's As You Like It. This play was selected because of its thematic relevance to children's self-concept, its developmental appropriateness for primary school children, and its feasibility in rural school settings. Specifically, the play addresses themes such as identity exploration, self-expression, role transformation, interpersonal interaction, and reconciliation, which are closely related to key dimensions of self-concept development. Its relatively clear plot, diverse characters, and movement from conflict to resolution also make it suitable for classroom-based drama activities that support safe emotional expression, imaginative participation, and peer interaction. In addition, the play's flexible structure makes it well suited to role-play, improvisation, group creation, and reflective discussion. Given the limited arts education resources in rural schools, As You Like It was considered especially appropriate because it can be implemented primarily through body movement, spoken language, and collective interaction rather than reliance on stage equipment.

To adapt the original play for rural primary school children, the research team simplified the plot, localized the language, and incorporated culturally familiar elements while retaining themes and situations relevant to self-concept development. Complex subplots and content less suitable for children's developmental level were removed, and the source text was reorganized into structured classroom activities centered on role-play, improvisation, small-group collaboration, and guided reflection. The final program was developed as a replicable teaching package, including an adapted script, activity flow sheets, facilitator prompts, and a classroom materials kit.

As summarized in Table 1, the intervention followed a process drama approach and integrated strategies such as role-play, improvisation, forum theater techniques, and peer feedback. Each unit was organized using a relatively fixed four-part structure: (1) situational introduction and warm-up, (2) role construction and plot development, (3) small-group collaboration and conflict resolution, and (4) whole-group sharing and reflective closure. The program lasted 10 days, approximately 7 h per day (with a 10-min break after each 60-min activity block), and was delivered in a rehearsal-room setting. For broader implementation, the structure can be compressed into typical class periods (e.g., 40–90 min) and embedded modularly into language arts, arts education, or integrated practice curricula.

**Table 1:** Pathway of intervention implementation.

Phase	Time	Goal	Key steps (in order)	Roles	Data collected	Expected outcomes (PHCSS)
0. Preparation (T0)	1 day before/ same day	Ethics and T0 baseline; norms; materials ready	Collect guardian and school consent; short volunteer training; administer PHCSS-T0 and demographics; prepare props and paper-theater materials	Teachers: planning and task allocation; Volunteers: note-taking and order maintenance	PHCSS-T0; class roster	Verified baseline, enabling later attribution of changes.
1. Introduction & Role awareness	D1–D2	Build safety and role awareness; stimulate interest	Warm-ups and rules; localized story synopsis; process drama (role try-on, improvisation); peer feedback	Teachers: model feedback; Volunteers: observation records	Observation sheets D1–D2	Greater safety and belonging; clearer self-representation; expected gains in Popularity and School Status.
2. Expression & peer mirroring	D3–D4	Practice expression and emotion recognition; experience being seen and needed	Voice–movement–presence drills; rehearse scene excerpts; structured peer feedback	Volunteers: guide feedback that is specific, respectful, and suggestive	Observation sheets D3–D4	Improved emotion recognition and expression; stronger perceived acceptance; reduced Anxiety.
3. Deepening roles & emotion regulation	D5–D7	Strengthen collaboration and emotion regulation within a full narrative	Discuss role goals, obstacles, and strategies; role swapping; practice self-soothing in high-arousal scenarios	Teachers: goal-oriented prompts; Volunteers: annotate regulation strategies	Observation sheets D5–D7	Higher self-efficacy; better emotion regulation; increased Happiness and Satisfaction.
4. Social reinforcement (Rehearsal/ Showcase)	D8–D9	Validate skills in a visible setting; obtain external support	Safety reminders and performance etiquette; costume rehearsals; small-scale showcase; structured audience feedback	Teachers: pacing and stage management; Volunteers: emotional buffer	Audience feedback records; observation sheets D8–D9	Stronger sense of being needed and belonging; gains in Popularity and Happiness.
5. Review & transfer (T1)	D10 + 1–2 days	Internalize experiences and transfer strategies; complete endline assessment	Critical-moment timeline review; small-group reflective writing; peer reading and encouragement; closing ritual; PHCSS-T1; sampled interviews (about 20 students)	Teachers: guide narrative integration; Volunteers: assessments and interview notes	PHCSS-T1; interview audio recordings; summary logs	Positive self-appraisal; consolidation of skills; cross-domain improvements from T0 to T1.

The teaching team included eight teachers with experience in drama in education and 30 trained volunteers. Teachers led sessions based on a shared manual and made in-the-moment adjustments as needed. Volunteers supported classroom management, documentation, individual support, and emotional accompaniment according to a predefined division of labor. Prior to implementation, all facilitators completed centralized training covering the script, session flow, and core prompting questions to promote consistency across sessions and fidelity to key components.

During implementation, students engaged in role-taking, scenario enactment, and collaborative problem-solving. Through “becoming someone else,” students externalized and reworked their emotions, relationships, and choices, gradually experiencing a process of understanding the self through role engagement, which was expected to support self-concept development as well as peer relationships and a sense of social belonging.

### 2.4 Measures

A Simplified Chinese PHCSS-2 was used, translated and back-translated following standard procedures and revised for Chinese child and adolescent samples. Prior work has reported acceptable internal consistency, test–retest reliability, and a six-factor structure in Chinese school-age samples, supporting its use for group comparison and screening (39–42). Because the study aimed to evaluate intervention effects rather than to re-validate the instrument, we treated the PHCSS-2 as an established measure and did not repeat exploratory or confirmatory factor analyses.

The PHCSS-2 was administered at T0 and T1 in both groups. Six subscale scores and the total score were computed following the manual for within-group pre–post change and between-group effect estimation. To evaluate internal consistency in the current sample, Cronbach's alpha coefficients for the total scale and each subscale were calculated using T0 data and reported in the Results.

### 2.5 Data analysis

#### 2.5.1 . Quantitative data analysis

First, questionnaire data were screened and cleaned; invalid questionnaires and cases with substantial missingness were excluded. Participants were assigned unique IDs and coded by group (0 = control; 1 = intervention). All statistical tests were two-tailed with α =.05.

Internal consistency reliability of the PHCSS-2 was examined using pretest (T0) data by calculating Cronbach's alpha for the total score and each subscale (Behavioral Adjustment, Intellectual and School Status, Physical Appearance and Attributes, Freedom from Anxiety, Popularity, and Happiness and Satisfaction).

Descriptive statistics (means and standard deviations) for pretest and posttest total and subscale scores were computed and reported by group. Baseline comparability between groups on pretest self-concept was assessed using an independent-samples *t-*test with the pretest total score as the outcome and group as the predictor.

To estimate the overall intervention effect, a one-way analysis of covariance (ANCOVA) model was specified with posttest PHCSS-2 total score as the dependent variable, group as the independent variable, and pretest total score as the covariate. Prior to ANCOVA, assumptions were evaluated, including linearity between pretest and posttest scores, homogeneity of regression slopes across groups, and homogeneity of error variances via Levene's test. The primary parameter of interest was the group main effect, indicating whether posttest scores differed significantly between groups after adjusting for baseline. Partial eta squared (partial η^2^) was reported as the effect size.

To describe within-group change in the intervention group, paired-samples *t-*tests compared pretest and posttest total scores. Where appropriate, effect sizes were estimated using Cohen's d (standardized mean difference) for paired change.

#### 2.5.2 . Qualitative data analysis

Classroom observations, volunteer logs, small-group discussion notes, student reflective writings, and interview transcripts were analyzed using thematic analysis following the phases described by Braun and Clarke. Before formal coding, the research team repeatedly read the materials and wrote analytic memos to develop familiarity with the corpus and reflect on emerging interpretations and assumptions. Meaning units served as the basic coding unit, and an iteratively updated codebook was used to support analytic organization and transparency across data sources.

Initial codes were generated across the full dataset and compared within and across sources. Related codes were then clustered into candidate themes through constant comparison. During this process, attention was paid not only to recurrent content, but also to what these patterns suggested about the social, emotional, and interactional processes through which changes in self-concept were experienced and narrated. Themes were further interpreted using a heuristic context-action-outcome lens, with particular attention to peer mirroring, emotion regulation, and role-based reflection as potential mechanisms.

Candidate themes were subsequently reviewed, refined, merged, or separated through iterative within-case and cross-case comparison until a coherent thematic structure was established. Final themes were retained when they were conceptually coherent, analytically distinct, relevant to the qualitative research questions, and supported by multiple excerpts and/or data sources. The credibility of the analysis was strengthened through triangulation across data sources and peer debriefing within the research team. Representative coded excerpts are provided in Appendix S3.

#### 2.5.3 . Mixed-methods integration

In the Results, we constructed a joint display in which core questions and focal topics were organized as rows and multiple columns juxtaposed: (a) quantitative evidence from PHCSS-2 change scores (T1 – T0) and distributional indicators, (b) qualitative evidence on mechanisms derived from thematic analysis, (c) integrated interpretations, and (d) practice implications. This parallel presentation enabled systematic examination of convergence, complementarity, and discrepancy between quantitative and qualitative findings, supporting mixed-methods meta-inferences. In particular, the joint display aligned the three hypothesized mechanisms—peer mirroring, emotion regulation, and role-based reflection—with specific patterns of change on the PHCSS-2. Narrative vignettes of selected cases were also used to concretize integrated patterns, enhance ecological validity, and clarify plausible pathways through which drama in education may influence children's self-concept under specific contextual conditions.

## 3 Results

### 3.1 Primary outcome and reliability

**Figure 2:**
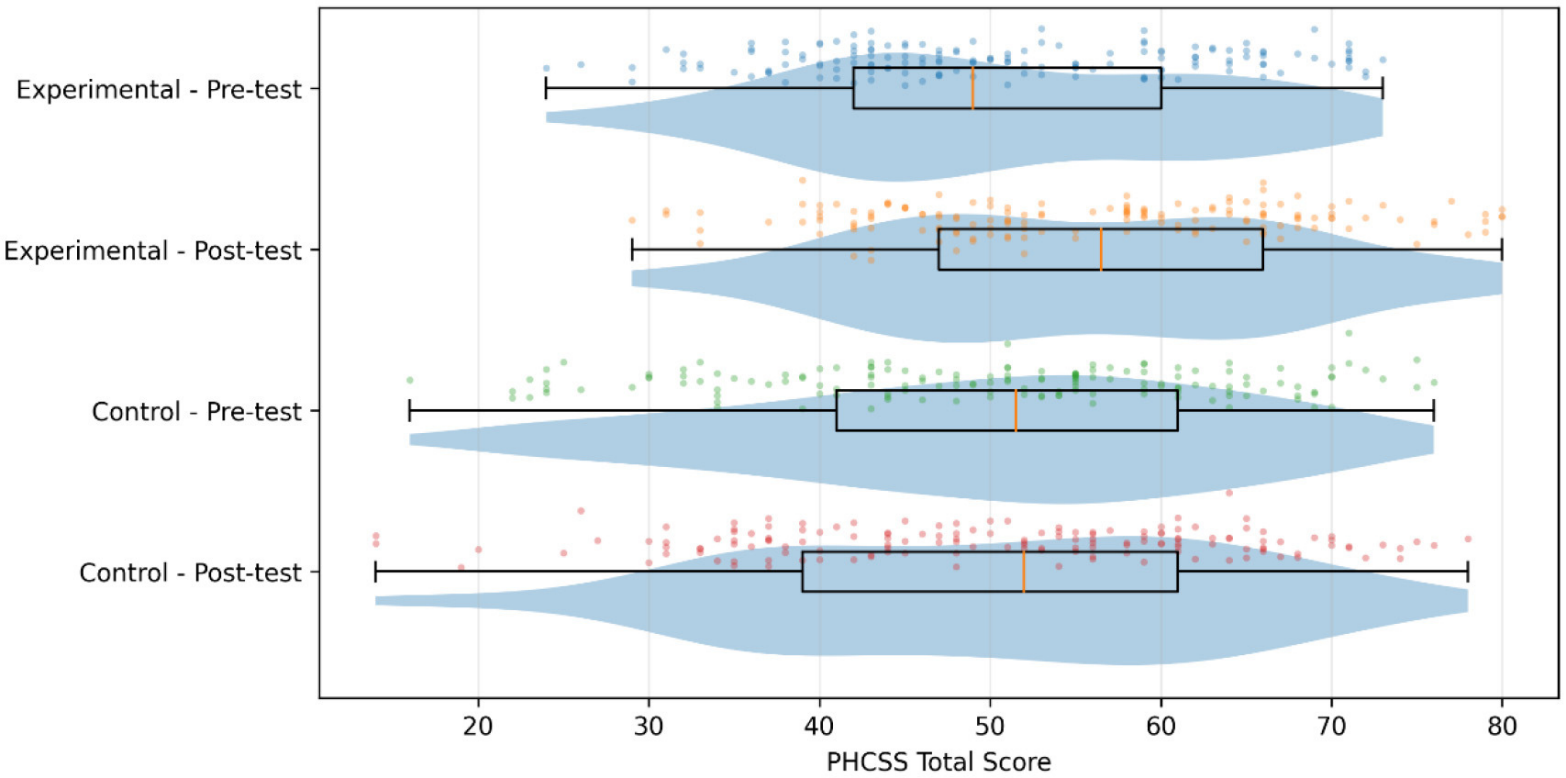
Raincloud diagram of pre- and post-test distributions for total PHCSS scores across two groups of children.

A total of 300 participants were included in the final analyses (intervention group, *n* = 150; control group, *n* = 150). At baseline (T0), the two groups had similar mean PHCSS-2 total scores (intervention: M = 50.79, SD = 11.69; control: M = 50.25, SD = 13.69). At post-test (T1), the intervention group's PHCSS-2 total score increased (M = 55.89, SD = 12.09), whereas the control group showed minimal change (M = 50.33, SD = 13.86). To visualize score distributions and pre–post shifts, raincloud plots were generated (Figure 2). Overall, the intervention group distribution shifted upward from T0 to T1, while the control group distribution remained comparatively stable.

Internal consistency of the PHCSS-2 was examined using baseline (T0) data. Cronbach's alpha for the total scale was α =.899 (standardized α =.899; 80 items), indicating good internal consistency. Subscale alphas were: Behavioral Adjustment (α =.645; 16 items), Intellectual and School Status (α =.699; 17 items), Physical Appearance and Attributes (α =.690; 13 items), Freedom from Anxiety (α =.676; 14 items), Popularity (α =.580; 12 items), and Happiness and Satisfaction (α =.629; 10 items). Overall, subscale reliability was acceptable to moderate. The Popularity subscale showed relatively lower internal consistency, and interpretations involving that subscale should therefore be made cautiously.

### 3.2 Baseline comparison and ANCOVA

To examine baseline comparability in total self-concept, an independent-samples *t-*test compared PHCSS-2 total scores at T0. Levene's test was not significant, *F* = 2.723, *p* = 0.100, supporting homogeneity of variance. The group difference at T0 was not significant, *t*_(298)_ = 0.363, *p* = 0.717, with a trivial effect size (Cohen's d ≈ 0.04). These results indicate that the groups were comparable at baseline.

To evaluate the intervention effect while accounting for individual baseline differences, a one-way ANCOVA was conducted with T1 PHCSS-2 total score as the dependent variable, group (1 = intervention, 0 = control) as the independent variable, and T0 total score as the covariate. The covariate was a significant predictor of post-test scores, *F*_(1, 297)_ = 78.634, *p* < 0.001, ηp^2^ = 0.209. After adjusting for baseline, the main effect of group remained significant, *F*_(1, 297)_ = 15.802, *p* < 0.001, ηp^2^ = 0.051.

As shown in Table 2, the ANCOVA results and estimated marginal means revealed clear group differences after controlling for baseline self-concept scores. Estimated marginal means indicated that, holding the covariate at a T0 score of 50.52, the adjusted post-test mean for the control group was M = 50.45 [95% CI (48.59, 52.31)] and for the intervention group was M = 55.77 [95% CI (53.91, 57.63)]. The adjusted mean difference was 5.32 (SE = 1.34), *p* < 0.001. Taken together, these results suggest that the drama in education intervention was associated with a significant positive improvement in children's self-concept beyond baseline levels.

**Table 2:** ANCOVA and estimated marginal means: effects of drama-in-education intervention on post-test self-concept scores.

Source	df1	df2	F	p	Partial η^2 ^
Panel A. ANCOVA results					
Covariate: pre-test total	1	297	78.634	< 0.001	0.209
Group (experimental/ control)	1	297	15.802	< 0.001	0.051
Error		297			
**Group**	**Adjusted M**	**95% CI (Lower)**	**95% CI (Upper)**
Panel B. Estimated marginal means of adjusted post-test			
self-concept scores (covariate set at M_pre_ = 50.52)			
Control	50.45	48.59	52.31
Experimental	55.77	53.91	57.63
Experimental – control	5.32	—	—

Additional test for group difference: mean difference = 5.32, SE = 1.34, *p* < 0.001. The dependent variable was the post-test total self-concept score; group (experimental vs. control) was the independent variable, and the pre-test total score was entered as a covariate. Partial η^2^ represents effect size; a value of 0.051 indicates a medium-sized main effect of group.

**Figure 3:**
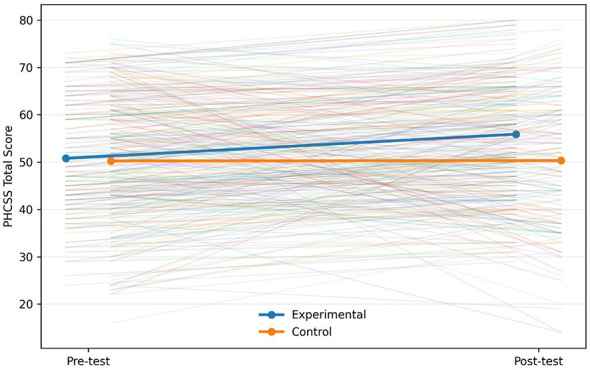
Individual change trajectories of PHCSS total scores for two groups of children.

Beyond overall distributional change, individual-level heterogeneity was examined using spaghetti plots (Figure 3). Most intervention participants showed upward trajectories from T0 to T1, but some individuals showed no change or decreases, indicating heterogeneity in response. In the control group, trajectories were flatter overall with minimal mean change.

### 3.3 Paired-samples *t*-tests and change-score visualizations

Prior to paired-samples *t*-tests, normality of pre–post difference scores were examined separately by group. As shown in Table 3, skewness and kurtosis for both groups' difference scores were near zero. Kolmogorov–Smirnov and Shapiro–Wilk tests were non-significant, suggesting that difference scores were approximately normally distributed and suitable for paired-samples *t-*testing.

**Table 3:** Pre- and post-test self-concept scores and within-group paired *t*-tests.

Group	*N*	Pre-test M ±SD	Post-test M ±SD	ΔM (Post – Pre)	t (df)	*p*	Cohen's d
Experimental	150	50.79 ± 11.69	55.89 ± 12.09	5.11	−14.18 (149)	< 0.001	1.16
Control	150	50.25 ± 13.69	50.33 ± 13.86	0.07	−0.05 (149)	0.961	≈ 0.00

ΔM, post-test minus pre-test. *t*, paired-samples t statistic within each group; Cohen's d, effect size for the paired comparison.

The negative *t* values reflect that the difference in SPSS was defined as pre-test minus post-test.

In the intervention group, PHCSS-2 total scores increased from T0 (M = 50.79, SD = 11.69, *n* = 150) to T1 (M = 55.89, SD = 12.09, *n* = 150). The mean increase was approximately 5.11 points, and the pre–post difference was significant, t (149) = 14.18, p < .001, with a large effect (Cohen's *d* = 1.16). This indicates a substantial within-group improvement in self-concept following the intervention.

In the control group, PHCSS-2 total scores changed minimally from T0 (M = 50.25, SD = 13.69) to T1 (M = 50.33, SD = 13.86). The mean difference was 0.07 points and was not significant, *t*_(149)_ = 0.05, *p* = 0.961, with a near-zero effect (Cohen's d ≈ 0.00), suggesting stability in self-concept in the absence of the drama in education program.

**Figure 4:**
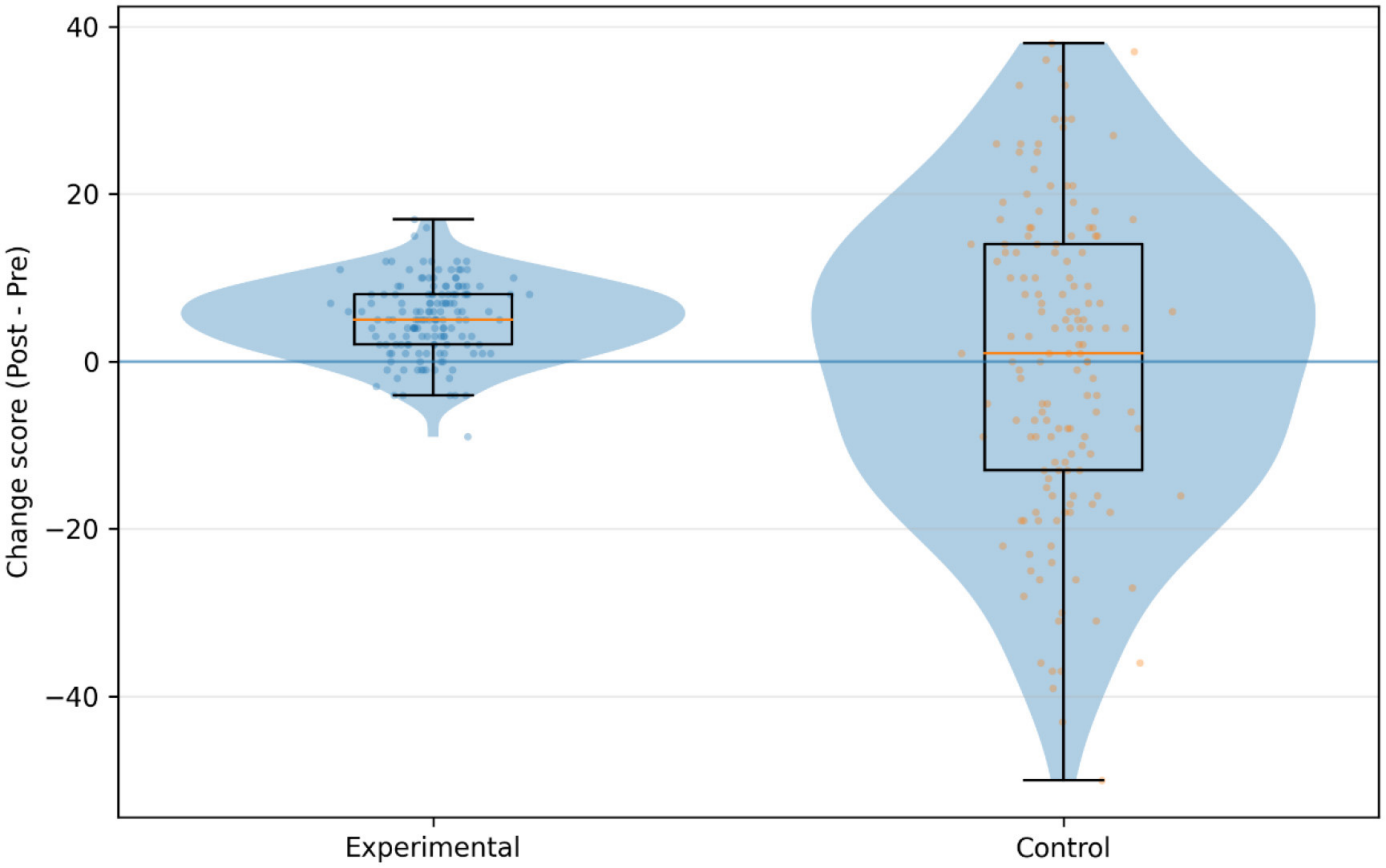
Comparison of the distribution of changes in total PHCSS scores (Δ = Post – Pre) between the two groups of children.

To compare net change during the intervention period, change scores were computed (Δ = T1 – T0) and visualized using violin and box plots (Figure 4). The intervention group's change-score distribution was predominantly above zero, indicating that most participants improved, whereas the control group's distribution clustered around zero, indicating little change. These visualizations were consistent with the inferential results.

Given the overall pattern of improvement and evidence of heterogeneity, the next section integrates interview and classroom observation data to examine children's lived experiences and the processes through which self-concept changes were experienced and articulated.

### 3.4 Qualitative themes

#### 3.4.1 Theme 1. Role experience and identity transformation

Within a structured rehearsal context, students gained mastery through manageable tasks and gradually developed a sense of being recognized through attention and affirmation from peers and teachers. Over time, students showed a tendency to seek more challenging tasks and to construct competence-oriented self-narratives. They often described breakthroughs via changes in voice and bodily expression. One student noted that after projecting their voice, they realized they could speak in a new way. Another attributed change to role demands that prompted action, explaining that as Orlando they had to speak first, so they initiated the first line (P7, post-session interview). During rehearsal, some students voluntarily took on higher-demand tasks, such as requesting more difficult lines and proposing adjustments to movement. In making sense of these shifts, one student framed the role as permission and psychological support, stating that it was not them becoming the character, but rather the character making them braver (P10, observation note). Overall, the theme captures a process of movement from passive compliance to active responsibility driven by role-based tasks, accompanied by renewed articulation of perceived competence. This theme indicates that children's self-concept change was closely tied to role-based experiences of competence and recognition. In relation to RQ2, children's narratives suggest that drama activities supported self-concept not merely through emotional expression, but by enabling them to experience themselves as capable, agentic, and socially acknowledged participants.

#### 3.4.2 Theme 2. Gendered expression and emotional freedom

Clear permission for expression and the establishment of safety boundaries appeared to reduce shame and loosen gender stereotypes, making students more willing to attempt more externalized emotional expression and more flexible role presentation. This theme emphasizes expansion in expressive range and temporary suspension of self-consciousness, rather than simple increases in emotional intensity. During activities, students demonstrated a variety of observable expressive strategies, such as resetting after crying, cross-gender role-taking, and exaggerated performance. These performances broadened expressive range and were associated with increased confidence. Students' accounts suggested that such attempts were most likely to occur in interactional climates where rules were clear and ridicule was not tolerated, which in turn supported sustained engagement in subsequent rehearsal and performance. The theme indicates that, under safety boundaries, students' space for experimentation in emotional expression and role presentation expanded, resulting in more diverse expressive forms. This theme indicates that the intervention contributed to self-concept by widening the range of socially acceptable self-expression under conditions of psychological safety. In this sense, self-concept change appeared to arise not from emotional release alone, but from a structured reduction in shame and self-concept that enabled more confident role-taking and participation.

#### 3.4.3 Theme 3. Collaborative interaction and social belonging

In collective rehearsal, peer prompting and positive feedback formed a stable mutual-support structure. Students not only completed tasks but also experienced a sense of being needed and were more likely to find a place within the group. The theme includes both specific interactional strategies and the way group feedback sustained willingness to participate. For example, peers developed prompt routines, such as pausing before speaking, to support line transitions and emotional entry. Group applause after performances occurred repeatedly and became an important cue through which students perceived acceptance and affirmation. Teacher observation notes further indicated that from Day 3 onward, some students began to actively seek more central roles, with richer emotional and physical expression. Overall, the theme illustrates how collaboration and group feedback provided cues of belonging during rehearsal and co-occurred with shifts in students' engagement posture. This suggests that drama-in-education supported self-concept not only through individual performance experience, but also through repeated interpersonal cues that students were accepted, needed, and able to contribute within the group. In relation to the research question, the theme indicates that changes in self-concept may partly emerge through strengthened social belonging, peer affirmation, and increased confidence in participation.

## 4 Discussion

### 4.1 Primary findings

The findings indicate that the drama-in-education programme was associated with short-term improvement in children's self-concept, with three proximal pathways helping to interpret this pattern. Across analytic approaches, results converged: the intervention group increased in PHCSS-2 total scores from pretest to posttest, whereas the control group remained stable. After adjusting for baseline self-concept, the group effect on posttest scores remained significant, with an adjusted between-group difference of 5.32 points (*p* < 0.001). Distributional visualizations were consistent with these findings, showing an upward shift and predominantly positive change scores in the intervention group.

Within-group change in the intervention group (*d* = 1.16) exceeded the adjusted between-group effect (ηp^2^ = 0.051), reflecting conceptually different quantities: change relative to the intervention group's own baseline vs. net differences between groups after baseline adjustment. Individual trajectories also indicated heterogeneity, with a minority showing no improvement or declines. Integrating quantitative patterns with qualitative themes, three proximal pathways may help interpret these changes. This mechanism-oriented interpretation is consistent with recent calls in arts education research to link specific arts practices in specific contexts to specific domains of socioemotional development, rather than making broad claims about generic arts benefits (43). A joint display (Table 4) was constructed to integrate the quantitative findings with qualitative mechanisms and practice implications.

**Table 4:** Joint display integrating quantitative effects and qualitative mechanisms of the drama-in-education intervention on children's self-concept.

Analysis focus	Quantitative results	Qualitative themes and mechanisms	Integrated interpretation	Implications for practice
Baseline comparison	Experimental and control groups had comparable pre-test self-concept scores (M = 50.79, SD = 11.69 vs. M = 50.25, SD = 13.69, *p* = 0.717).	Initial student self-reports indicated no major differences in self-concept prior to intervention.	Quantitative results confirm qualitative observations of similar baseline self-concept across groups.	Intervention should be targeted at both groups, as they start with comparable levels of self-concept.
Post-test self-concept gain	Experimental group showed significant increase in self-concept post-test (M = 55.89, SD = 12.09), compared to a minor change in control group (M = 50.33, SD = 13.86).	Students reported feeling more confident, motivated, and willing to challenge themselves after the drama intervention.	Convergence: both quantitative data and qualitative reports support a substantial self-concept gain in the experimental group.	Focus on maintaining engaging and challenging tasks for the experimental group, emphasizing self-reflection through roles.
Effect size of intervention	Cohen's d = 1.16 for experimental group (significant effect); Cohen's *d* = 0.00 for control group (no effect).	Students stated they felt braver and more capable after participating in difficult scenes and roles.	Quantitative results (large effect size) match the qualitative findings of increased self-efficacy, especially in the experimental group.	Focus interventions on roles that challenge students' perceived capabilities.
Impact on emotional expression	No significant differences in emotional expression dimensions (e.g., anxiety); minimal improvement in the control group.	“Crying and returning to a neutral state,” “cross-gender role play,” and “exaggerated performances” expanded emotional expression and boosted confidence.	Qualitative data explain that while the overall anxiety levels didn't change drastically, the emotional expression significantly improved for the experimental group, particularly in role-play.	Integrate more opportunities for emotional expression, especially through role-playing that pushes emotional boundaries.
Peer interaction & social belonging	ANCOVA shows significant group differences in social self-concept, with experimental group outperforming control group.	Peer feedback and group support (e.g., applause after performance) helped build a sense of belonging. Students felt “needed” in their group roles.	Strong alignment between quantitative social self-concept improvements and qualitative reports of enhanced group belonging and peer interaction.	Promote collaborative tasks that reinforce peer recognition and team-based outcomes.
Gender expression & identity	Minor difference in self-concept dimensions related to gender expression and identity.	Drama encouraged students to explore different gender roles and step outside traditional boundaries, reducing shame and stereotypes.	Gender expression and role flexibility may contribute to increased self-confidence, though the overall quantitative impact on gender dimensions was modest.	Incorporate activities that challenge traditional gender norms and encourage diverse forms of self-expression.
Group comparison on post-test scores	After controlling for pre-test scores, experimental group showed significant increase in post-test scores (M = 55.77).	Students in the experimental group noted feeling “braver” and “more outspoken” in class, with visible role-based behavioral changes.	Both quantitative and qualitative data suggest a clear positive effect of the drama intervention on self-concept, especially in terms of social courage and class participation.	Continue fostering an environment where children can experience growth in self-efficacy through role-playing and public performance.

First, role experience and identity transformation suggested competence-related reappraisals. This interpretation is in line with drama education research suggesting that role-taking, together with sensitive teacher encounters, can enable children to learn safely through trial and error (44). Under structured tasks and progressive rehearsal, children described or displayed increased willingness to initiate speech, project voice, and assume responsibility, consistent with short-term gains in overall self-concept. Second, collaborative interaction and social belonging provided social cues for self-evaluation: peer prompting, positive feedback, and group affirmation supported feelings of being needed and accepted, aligning with predominantly positive change distributions. This emphasis on belonging is consistent with research defining school belonging as feeling accepted, respected, included, and supported, as well as with studies linking peer acceptance to more positive self-perceptions and better adjustment in children (45–47). Third, expanded emotional and gendered expression under safety boundaries appeared to reflect increased expressive range and participation space rather than a straightforward reduction in negative affect. Rather than treating expression as emotional release alone, this interpretation aligns with work showing that classroom emotions are shaped by emotionally expressive environments and relationships (48). Given that the quantitative evidence primarily supports total-score improvement and subscale reliability varied (notably lower reliability for Popularity), subscale-specific claims should be treated cautiously. A conservative interpretation is that improved expressive freedom and participation quality supported more positive self-descriptions, reflected in higher overall self-concept.

Overall, the mechanism account is intentionally cautious. Structured role tasks may provide mastery experiences, group processes may supply social-evaluative cues, and explicit safety boundaries may reduce barriers to expression and sustain engagement. These interpretations are consistent with the observed pattern of results, but they do not replace direct measurement of mediators.

### 4.2 Contextual fit and implementation implications

This study took place in a rural primary school context where classroom order and collective coordination are salient norms. The observed heterogeneity suggests that for a minority of children, self-presentation demands and evaluative pressure may be a risk point. Implementation priorities should therefore emphasize controllability and psychological safety rather than simply increasing exposure intensity. This emphasis on controllability and psychological safety is consistent with evidence that school belonging may help reduce the impact of negative school climate on students' mental health (49).

A feasible approach is to begin with low-risk, structured units and gradually increase improvisational demands under clearly articulated rules. The qualitative themes indicate that safety boundaries and peer support are practical prerequisites for expressive expansion and sustained participation. For students showing greater volatility, tiered task difficulty, role rotation, and intentional peer pairing may reduce self-presentation burden. Localizing canonical texts for children and embedding local language, rural-life references, and intangible cultural heritage elements may increase acceptability and participation quality while preserving classroom order. These implications are grounded in the observed variability rather than in general advocacy for arts education.

These findings may be particularly meaningful in educationally underserved rural school settings, where structured opportunities for psychosocial support, recognition, and school-based mental health promotion are often more limited than in better-resourced schools. In this context, the observed gains in self-concept appear to have been supported by low-cost classroom processes, including role-based experiences of competence, peer affirmation, and psychologically safe opportunities for self-expression. Because this study did not include an urban comparison group, these findings should be interpreted as context-specific evidence from a rural setting rather than as proof of urban–rural differences.

### 4.3 Emotional expression, role reflection, and self-concept

Taken together, the findings suggest that drama in education may support self-concept not primarily through emotional catharsis, but by establishing classroom conditions in which expression can occur, be socially held, and be meaningfully integrated. This interpretation is compatible with creative arts therapies models in which change arises through safe externalization, reflection, and meaning-making rather than simple discharge of feeling (50, 51).From role-theoretical perspectives, role entry can provide permission to try atypical expressions within a lower-risk frame; reflective closure supports movement between role and self; and collective rehearsal offers ongoing affirmation and cues of belonging. These processes are proposed as plausible pathways aligned with the observed total-score change, while acknowledging that mediators (e.g., emotion regulation, self-efficacy) were not directly measured.

An art-therapy-informed lens may further inform school-based program design. While drama in education and arts therapies differ in disciplinary aims, both emphasize externalization, meaning-making, and relational holding. Brief post-scene integration steps (e.g., short drawing, symbolic representation, or brief writing followed by small-group sharing) may help consolidate emotional naming and learning without shifting the program into a clinical frame.

## 5 Limitations and future directions

This quasi-experimental study examined the short-term effects of a drama in education intervention on self-concept among rural Chinese primary school children. Participation in a short, high-intensity program was associated with a significant improvement in PHCSS-2 total scores, supported by qualitative evidence indicating enhanced self-expression and social belonging. Across data sources, findings suggest that structured role-taking within clear safety boundaries, combined with reflective closure, may help children rehearse and consolidate more positive self-narratives. By integrating a child-appropriate adaptation of a canonical text with localized language and cultural elements, the intervention demonstrates a feasible and potentially replicable arts-based pathway for school-based psychosocial support in low-resource settings.

Several limitations should be acknowledged. The quasi-experimental, single-school design cannot fully rule out maturation, history, or expectancy effects, and it constrains generalizability. The focus on short-term outcomes may underestimate emotion-related changes, as some subscales may be less sensitive and qualitative improvements in expressive quality may not be fully captured by brief self-report measures. In addition, reliance on child self-report as the primary outcome limits triangulation.

Future research should strengthen study rigor by incorporating randomization and longitudinal follow-up to model individual- and class-level developmental trajectories. Such comparative work would help determine whether the effects and mechanisms observed in this study are specific to educationally underserved rural settings or generalize across differently resourced school contexts. Measurement approaches would benefit from multi-informant indices (e.g., teacher or caregiver reports), standardized behavioral observations, and feasible physiological indicators. From an implementation science perspective, systematic documentation of intervention fidelity and comparative testing of localized variants are needed to define minimum viable inputs and inform scalability. Overall, the present mixed evidence supports a stable short-term association between drama in education and improved self-concept, while longer-term maintenance, causal strength, and large-scale applicability remain priorities for higher-rigor investigation.

## Data Availability

The original contributions presented in the study are included in the article/Supplementary material, further inquiries can be directed to the corresponding author.
