# Supplementary material for: Drama-in-education in rural Chinese primary schools: effects on self-concept, social connectedness, and psychosocial wellbeing
Source: Front Public Health. 2026 Apr 22;14:1812030. doi: 10.3389/fpubh.2026.1812030 (PMC13143897; doi:10.3389/fpubh.2026.1812030)
Supplement: Supplementary file 1 [file Supplementary_file_1.docx]

**Appendix S1**

**Piers–Harris Children’s Self-Concept Scale, Second Edition, PHCSS-2**

Below are 80 questions that ask how you see yourself. Please decide which questions fit your actual situation and which do not. If you think a question applies to you or mostly applies to you, circle “Yes” next to the corresponding number on the answer sheet. If it does not apply to you or mostly does not apply to you, circle “No” You can only answer each question once, and every question must be answered. Please note that you are asked to answer according to what you truly believe about yourself, not how you think you should be. Please do not make corrections on the answer sheet. After filling it out, please return the sheet along with this form.

1. My classmates make fun of me.
2. I am a happy person.
3. I find it hard to make friends.
4. I am often sad.
5. I am smart.
6. I am shy.
7. I feel nervous when the teacher calls on me.
8. I am bothered by my appearance.
9. I will become an important person when I grow up.
10. I get anxious when school exams are coming up.
11. I don't get along with others.
12. I perform well at school.
13. When something goes wrong, it is often my fault.
14. I bring trouble to my family.
15. I am strong.
16. I often have good ideas.
17. I am an important member of my family.
18. I often want to do things my way.
19. I am good at doing crafts.
20. I give up easily.
21. I do well with my schoolwork.
22. I do many bad things.
23. I am good at drawing.
24. I am good at music.
25. I don't perform well at home.
26. I take a long time to finish my schoolwork.
27. I am an important person in my class.
28. I get nervous easily.
29. I have beautiful eyes.
30. I can speak well in front of the whole class.
31. I am a dreamer at school.
32. I often tease my siblings.
33. My friends like my ideas.
34. I often get into trouble.
35. I am obedient at home.
36. I am lucky.
37. I often worry.
38. My parents have too high expectations for me.
39. I like to do things my way.
40. I feel like I am always losing things.
41. I have good hair.
42. I volunteer to do things at school.
43. I wish I were different from others.
44. I sleep well at night.
45. I hate school.
46. I am often one of the last people picked in games.
47. I often get sick.
48. I am often stingy with others.
49. My classmates think I have good ideas.
50. I am unhappy.
51. I have many friends.
52. I am happy.
53. I don’t express opinions on most things.
54. I am beautiful.
55. I am energetic.
56. I often fight.
57. I get along well with boys.
58. Others often make fun of me.
59. My family is disappointed in me.
60. I have a pleasant face.
61. I always feel something goes wrong when I try to do something.
62. I am often teased at home.
63. I am a leader in games and sports activities.
64. I am clumsy.
65. In games and sports, I just watch but don't participate.
66. I often forget what I have learned.
67. I get along well with others.
68. I get angry easily.
69. I get along well with girls.
70. I like reading.
71. I prefer doing things alone rather than with many people.
72. I like my siblings.
73. I have a good body.
74. I am often afraid.
75. I always break things or damage things.
76. I can be trusted by others.
77. I am different from others.
78. I often have bad thoughts.
79. I cry easily.
80. I am a good person.

**Appendix S2**

**Interview Outline**

**Objective of the Interview:**

1.Explore students' emotional experiences in educational drama activities.

2.Understand how students experience changes in self-concept through role-playing and situational enactment.

3.Assess the impact of educational drama on students' social interactions, confidence, and emotional regulation.

**I. Background Questions**

1. Please briefly describe the drama activity you participated in.
2. Have you had any prior experience with drama or performance? If so, how did those experiences impact you?
3. What motivated you to participate in these drama activities? What did you hope to gain from them?

**II. Self-Perception and Changes**

1. Before starting the drama activities, how did you feel about yourself in school? For example, did you feel confident when doing things?
2. After participating in the drama activities, do you feel like you've changed in any way? Do you feel more confident now?
3. During the drama activities, did you discover anything about yourself that you hadn’t noticed before? For example, did you do anything in the performances that surprised you?

**III. Emotional Expression and Emotional Regulation**

1. How did you express your feelings during the drama activities? Do you feel that performing allowed you to say things you normally wouldn’t say?
2. Was there a particular drama activity that made you feel really happy, excited, or nervous? How did you manage to calm yourself down when you felt nervous?
3. Do you feel like it’s easier to control your emotions during role-playing performances?

**IV. Relationships with Classmates**

1. When you performed with your classmates, did you feel supported or helped by them? How did you all help each other?
2. Do you think that practicing and performing together helped improve your relationship with your classmates? Why?
3. After participating in these activities, do you feel that your relationship with your classmates has changed? Do you feel more willing to talk or help others?

**V. Role and Identity Feelings**

1. What was your favorite role to play? Why did you like it? What made that role special?
2. When you were performing, did you feel like you became that character? Can you share any thoughts or feelings you had while being that character?
3. Do you think that playing a role made you feel braver or more confident? Can you give an example?

**VI. Final Questions**

1. Overall, what did you enjoy most about the drama activities? What did you gain from participating?
2. If you had the chance again, would you want to join similar drama activities? Why or why not?
3. What do you think these drama activities helped you with? For example, did they help you become more confident, improve your relationships with classmates, etc.?

**Appendix S3**

**S3.1 Open Coding Examples**

| **Original Excerpt** | **Meaning Unit Segmentation** | **Initial Codes** | **Code Merging/Revision** |
| --- | --- | --- | --- |
| “When I played the Duke, I needed to project my voice; later I realized I could actually speak like this.” | Voice projection; role requirement; self-discovery | Voice projection; modeling authority; emergent self | Role embodiment → sense of vocal agency |
| “I’m usually quiet, but as Orlando I had to speak first, so I said the first line.” | Quiet baseline; role requires opening line; actually opened | Temperament contrast; speaking first; initiating attempt | Role-driven → proactive opening |
| “After roles were assigned, I volunteered for a longer and harder part and suggested changing a movement.” | Role switching; volunteered for harder part; proposed modification | Escalating challenge; creative suggestion | Difficulty progression → identity expansion |
| “It’s not that I became the role; the role made me different—I felt braver.” | Role–self co-construction; ‘brave me’ | Identity restatement; brave self | Role transformation → self-reappraisal |
| “I even cross-dressed to play Rosalind—am I awesome or what?” | Cross-dressing; legitimation question | Gender boundary crossing; prop-enabled agency; norm confirmation | Flexible gender roles → license to express |
| “I cried for a moment. The teacher said it was okay—cry and continue—then I wasn’t afraid.” | Crying; permitted; continue; not afraid | Permission for emotional release; return to task | Sanctioned vulnerability → emotional reset |
| “During rehearsal I handed tissues to peers, patted shoulders, and cued lines.” | Handing tissues; soothing | Caregiver role; emotional tending | Care that crosses gender expectations → agentic caregiving |
| “Playing the strongman felt great. I could perform freely, and the teacher said it should be really exaggerated here.” | Free performance; license to exaggerate | High-intensity expression; validated exaggeration | Exaggerated expression → emotional liberation |
| “When I got stuck, he whispered ‘pause first, then speak,’ and I picked it up.” | Stuck; peer prompt; rejoined | Peer cue; task resumption | Micro-intervention → performance recovery |
| “Everyone looked at me and waited for my line—I felt they needed me.” | Waiting for my line; being needed | Being seen/needed; ring-shaped structure | Collective waiting → belonging affirmed |
| “After the performance everyone applauded; I felt I had a place in this group.” | Applauded; having a place | Positive peer feedback; recognition of role position | Positive feedback → belonging confirmed |

**S3.2 Axial Coding Examples**

| **Category (Theme)** | **Subcategory** | **Context** | **Actions** | **Outcomes** |
| --- | --- | --- | --- | --- |
| Role Experience & Identity Transformation | Role-driven behavioral boundary-crossing and identity reappraisal | Role switching; high-demand lines; teacher modeling with stepwise rehearsal; small-group showcasing | Initiate speech, amplify voice, take on harder roles, propose line changes | Courage/self-efficacy ↑; “I can do it” competence representation ↑; identity narrative updated |
| Gender Expression & Emotional Freedom | License to express | Explicit rules of “permission to exaggerate” and “vulnerability can be held”; flexible role gendering; mutual helping encouraged | Exaggerated expression, return to task after crying, handing tissues, shoulder pats, cross-dressing | Shame ↓; expressive freedom ↑; gender-role elasticity ↑ |
| Cooperative Interaction & Social Belonging | Peer prompts and group rules enable completion and belonging | Waiting for peers’ lines; peers whisper prompts for actions/lines | Smoothly picking up lines; collective waiting and applause feedback | Completion rate ↑; felt needed/has a place ↑; belonging ↑ |

**S3.3 Selective Coding Examples**

| **Core Category** | **Path/Theme** | **Storyline** |
| --- | --- | --- |
| Educational Drama Activates Self-Concept | Path 1: Role Experience & Identity Transformation | In role-switching contexts, children initiate speech, project voice, take on harder tasks, and deliver key lines; visible success advances “I can do it” competence representations and identity updating. |
|  | Path 2: Gender Expression & Emotional Freedom | When the classroom makes expression license explicit, role gender is flexible, and peers are accepting, children can exaggerate performance and “reset after crying,” reducing shame and increasing expressive freedom. |
|  | Path 3: Cooperative Interaction & Social Belonging | Peer prompts support children to “pause then speak” and pick up lines during hiccups; waiting and applause confirm being needed and one’s place, enhancing belonging. |
